# The ‘Will of the People’: The Populist Challenge to Democracy in the Name of Popular Sovereignty

**DOI:** 10.1177/09646639231153124

**Published:** 2023-01-29

**Authors:** Oliver Schmidtke

**Affiliations:** Centre for Global Studies, 8205 University of Victoria, Canada

**Keywords:** populism, democracy, popular sovereignty, migration, borders, European Union, Germany

## Abstract

This article analyses how right-wing populist actors claim to represent the “voice of the people” and express “popular sovereignty” as a mode of challenging the traditional constitutional foundation of liberal democracy. This hypothesis is illustrated by an investigation into the political discourse of the *Alternative for Germany* considering how this populist actor has developed a political strategy claiming to speak for the “people” in an authentic and immediate fashion. The analysis of this actor's political mobilization shows how the championed direct democratic representation is couched in a sovereigntist discourse that relies on divisive identity markers rather than genuine democratic participation. Drawing on Carl Schmitt's concept of the political, the article interprets right-wing populism as invoking a permanent “state of exception” that employs an emotionally charged *friend*–*enemy* distinction whose logic of representing the people has the potential of triggering radical political change as well as undermining the integrity of rule-based democracy.

## Introduction

Judging from their political rhetoric, one could perceive populist movements as guardians of the essential constitutional order underpinning democracy. With its claim to be the direct and genuine *vox popoli*, the voice of the people, populism evokes the fundamental principles on which modern democracy rests (Espejo, 2017). At the core of the populist challenge to established political actors and institutions is the claim that the “will of the people” has lost its foundational role in guiding government practices. In this respect, the mobilizing idea behind the populist resurgence across Europe and the Americas reflects a radical critique of the current constitutional order and a plea for empowering the “people” depicted as disenchanted and deprived of their legitimate role. Some commentators have accurately portrayed populism as a political force that mirrors the deteriorating trust in current democratic practices (Bang and Marsh, 2018; Kriesi, 2014; Mudde, 2007; Mudde and Kaltwasser, 2012). As the late Peter Mair diagnosed, the hollowing out of Western democracy has produced its own—democratically legitimated—nemesis (Mair, 2013). In a similar vein, Appadurai (2017) speaks of a widespread “democratic fatigue” providing the grounds for a fundamental challenge to the political status quo (similarly, Dawson and Hanley, 2016; Müller, 2011).

Yet, as recent developments for instance in Brazil, Hungary, or Poland have underlined, populism in its right-wing, nationalist iteration has the propensity of promoting authoritarian modes of government, undermining the integrity of the rule of law, and challenging the procedural framework of liberal democracy in a variety of facets (Antal, 2017; Bugaric and Kuhelj, 2018; Galston, 2018; Kelemen, 2017; Stavrakakis, 2014). The populist plea for empowering the “people” tends to promote practices in flagrant opposition to the foundational doctrines of liberal, rule-based democracy. In particular in cases where populist parties have captured positions of power, their plea for emboldening the disillusioned “people” has regularly morphed into government practices that violate traditional standards of democratic accountability (Albertazzi and Mueller, 2013; Blokker, 2021; Goodhart, 2017; Otjes and Louwerse, 2015).

Scholars have noted persistently the ambivalent impact that populist politics has on democracy (Kaltwasser, 2012; Katsambekis, 2017). In essence, they depict populism as a mode of political mobilization rather than a coherent political ideology with a blueprint on how to re-organize the political community (Moffitt, 2016). Populist political mobilization and its radical contestation of established power structures can be found on both the left and the right. Populism's defining mark is the challenge to the status quo, the invocation of the people deprived of its legitimate rights, rather than a coherent set of policies or government practices. Considering the ideologically evasive (Stanley, 2008) and context-specific nature of populism, this article raises the following questions: Are there elements in the nature of the populist mobilization that bring it into a fundamental, if not irreconcilable conflict with the constitutional order of liberal democracy (Pinelli, 2011)? More specifically, do episodic illiberal tendencies reveal an innate quality of populism in particular with respect to its right-wing, nationalist manifestation? Is there a core idea that drives and directs the populist challenge to liberal democracy?

The central hypothesis that this article seeks to advance is that, in spite of its powerful appeal to the democratic empowerment of the “people,” populism's political mobilization operates on a notion of the political that is intrinsically linked to authoritarian tendencies. Irrespective of its vague ideological commitments and designs for constitutional reform, populism employs modes of political mobilization that have a strong affinity to nondemocratic practices.

This article develops this argument in three steps. First, I explore the similarities between Carl Schmitt's notion of the political and the sovereign on the one hand, and the dominant forms of populist political mobilization and its plea for “popular sovereignty” on the other. This theoretically driven exploration will provide the conceptual framework for investigating the way in which populist movements from the right have promoted their distinct understanding of the “sovereign people.” Second, I investigate the way in which populist parties from the right have launched effective mobilizing campaigns and garnered considerable electoral support. The empirical study is based on a discourse analysis of the political mobilization conducted by the *Alternative for Germany* (AfD), the first right-wing, nationalist party elected to Germany's Federal Parliament since World War II.

The aspiration is both theoretical—investigating the ideological core of contemporary populism—and, with its interpretative focus on Europe and more specifically Germany, empirical in nature. The German context is particularly intriguing for studying populism as a fundamental challenge to the constitutional order of liberal democracies: In its 20th century past, the country has witnessed the rise of a dictatorial regime whose authority rested centrally on the claim to “empower” the German people. The Federal Republic of Germany's fundamental post-war commitment to protecting democracy has been to learn from the Third Reich and National Socialism. Yet, the rise of the AfD has underlined that German society is not immune to the lure of authoritarian politics and radical opposition against liberal democracy. In this respect, the article focuses on the rise of a populist party in a country that historically has a heightened sense of how vulnerable the democratic order is. Third, I analyze the AfD party's political campaigns as a way to revisit the more theoretically informed question regarding populism's understanding and impact of the democratic empowerment of the “people.”

## Conceptualizing the “Political”: The Perpetual State of Exception as Populism's Lifeblood

The reference to the “sovereign people” or “sovereignty” is a prominent feature in the political campaigns of populist actors. This rhetoric does not occur by accident. First, the claim to restore the “will of the sovereign people” speaks to the populist opposition to internationalization and globalization. The *America First, France First, Italy First, etc.* slogans conjure the notion of a nation-state with a clearly defined political community protected from the threats and uncertainty associated with a globalizing world. In this respect, the evocation of sovereignty as a mode of politically organizing the commons reflects the scholarly debate on the declining power of the nation-state and a political environment increasingly shaped by cross-border mobility and global markets. Discursively, contemporary populism invokes the idea of the territorially delineated nation-state demarcating the political community and promising protection to its citizens (Kallis, 2018; Schmidtke, 2022). Populist political campaigns tend to construct a dramatized contrast between the ostensibly simple and shielded world of the sovereign nation-state on the one hand and the uncertainty and threatening menaces associated with the international world on the other (see Stavrakakis et al., 2018). I will come back to this Hobbesian logic as a foundational feature of populist political mobilization in the following section.

Second, populists portray themselves as the guardians of the “will of the people” referencing the tradition of a less state-centric notion of “popular sovereignty” with an emphasis on political struggles and legitimacy. In the wake of the 2014 European elections, Marine Le Pen thanked her supporters in France by saying “the sovereign people have spoken loudly to say they want to be master of their own destiny.”^1^ Blühdorn and Butzlaff (2019: 194) speak of the “discursive arena for the performance of sovereignty” that populist actors employ in their political rhetoric in order to boost their democratic legitimacy and broader popularity (similarly Kelly, 2017). Populist parties mobilize the notion of popular sovereignty proclaiming to advocate for the silent majority. As their champion, the populist parties claim to provide representation to a polity deprived of their voice and the means to defend their fundamental interests.

In her analysis of popular sovereignty, Nootens underlines how this concept is historically tied to the process of democratization and democratic practice. Based on the notion of the “sovereign people,” the marginalized and excluded have been able to challenge those in power in the name of a fundamental democratic principle, namely the constituent power of the “people” as the ultimate sovereign. As Nootens (2013: 1) explicates, popular sovereignty emerged from “the defense of specific rights and interests and more generally the need to protect themselves against oppression by rulers and their intermediaries.” Historically, the excluded social groups have mustered “popular sovereignty” as a principle to delegitimize governments identified as betraying the democratic mandate of the “people.”

It is in this logic of “popular sovereignty” as a mode of political contestation and struggle that populist actors make explicit reference to an essential alienation from the polity and its modes of self-governance. Mair (2006: 25) speaks of “a notion of democracy that is being steadily stripped of its popular component—democracy without a demos.” The idea of “popular sovereignty” allows populists to challenge the political establishment postulating that those in power lack the proper consent of the citizens. In response, they use popular sovereignty as a political tool to extract rights and privileges from unresponsive elites. Such a basic democratic claim—the invocation of an ideal of popular sovereignty against what populists say are insufficient and improper means of holding rulers to account—grants legitimacy and appeal to the defining mark of populism. In this respect, the core populist claim is vested in an idea, “which pits a virtuous and homogeneous people against a set of elites and dangerous ‘others’ who are together depicted as depriving (or attempting to deprive) the sovereign people of their rights, values, prosperity, identity and voice” (Albertazzi and McDonnell, 2008: 3).

Populist actors mobilize the demand for “popular sovereignty” with the explicit intent of challenging established decision-making procedures in liberal democracies (Urbinati, 2019). Yet, it is not clear how this plea for radical change should inform and guide concrete constitutional reforms. In the case of contemporary populist parties in Western democracies, the rhetorical appeal to basic democratic principles does not necessarily translate into constitutional processes facilitating greater degrees of democratic participation and accountability in the liberal tradition. Rather, populists promise to advance a democratic practice that is radically different from the one guiding parliamentary democracy. In an unmediated way, the populist defence of popular sovereignty is to give voice to a united, homogenous entity called the “people.” Corrias (2016) describes this form of representation as a “politics of immediacy” that seeks to affirm the power of the “people” demonstrating their sovereign rights (for instance, at rallies, demonstrations, referenda, etc.). In this respect, manifestations and rallies are a distinguishing attribute of populist politics. They are not only modes of political mobilization but also an essential feature of how populists envision “popular sovereignty” in practice. In such performative political acts, populist parties commonly rely on their charismatic leader to stage the “will of the people” and provide them with a sense of tangible presence.

### Popular Sovereignty and the Friend-Enemy Distinction

In spite of making the notion of popular sovereignty the cornerstone of their political campaigns, populist parties do not promote a coherent proposal for constitutional reform—at least as long as they operate as an oppositional force. Attempts to identify a form of “Populist Constitutionalism” (Halmai, 2018) have indeed been largely futile (Corduwener, 2014). The pleas for democratic renewal are general in nature—mostly focused on forms of more direct democracy—and gain their thrust through the emphatic, albeit unspecific reference to the “people.” Still, it is worth considering how the radical challenge to state authorities in the name “ordinary people” informs and envisions constitutional reform. In the populist narrative of being deprived by political elites, how are the “people” meant to find a proper voice and have their interests safeguarded? How is the true sovereign expected to express its political preferences and how should democratic self-rule be organized? In short, what are the notions for empowering the “people” envisioned in the political mobilization of right-wing populist parties?

As will be explicated in the subsequent empirical section, the populist mode of advocating for the rights of the people has a strong affinity to authoritarian traditions. In its challenge to established institutions and procedures of liberal democracy, right-wing populism makes explicit reference to a superior legitimacy that rests outside of the procedural logic of the constitutional order. In this respect, Mudde (2021) speaks of populism as providing “an illiberal democratic response to undemocratic liberalism.” Very much in line with Schmitt's inter-war criticism of parliamentary democracy, populist mobilization evokes a notion of politics as a “state of exception,” a continuous existential crisis of the political community that cannot be addressed effectively with traditional rule-based, democratic procedures.

From a theoretical perspective, there is a salient similarity between the core ideas of the current wave of populist opposition to the liberal order and the critics of liberalism in the inter-war period. Most prominently, Carl Schmitt articulated the cornerstones of the latter position in his work on the crisis of parliamentary democracy (Schmitt, 1988) and the *Concept of the Political* (Schmitt, 2008). His notion of sovereignty and the political authority emanating from it can help to gain a better understanding of the contemporary populist challenge and its vision for constitutional change. What I contend is that Schmitt's concern for—as he puts it in the opening line of his book (Schmitt, 2008)—the essence of the “political” reflects the core of the populist challenge to the status quo of liberal democracy.^2^

One critical element of Schmitt's work as a constitutional and political theorist are his ideas about what constitutes a stable, functioning, and legitimate political order. In line with a Hobbesian reasoning, Schmitt posits that the legal framework for the political life of a community rests on a sovereign decision that legal norms and practices cannot generate themselves (Schmitt, 2005). Schmitt considers this sovereign authority as a precondition for a constitutionally designed legal order (Ingimundarson and Jóhannesson, 2020). As Cristi (1998: 198) observed, for Schmitt the “grounds of a constitution are existential.” According to Schmitt's theoretical framework developed in *Political Theology* (Schmitt, 2005), a constitutional order requires a consensus of the political community that a sovereign decision establishes responding to an extraordinary challenge threatening the community's integrity. Schmitt describes the essence of sovereignty “not as the monopoly of domination or coercion, but as the monopoly of decision” (Schmitt, 2005: 20). It is this reasoning that is reflected in Schmitt's famous dictum that the “sovereign is he who decides on the exception” (Schmitt, 2005: 5). It requires an extra-legal force to uphold a particular legal order or to establish a new one in a “state of exception.” The latter interpreted as a society deeply fragmented by social or ideological conflict and unable to defend itself against threats (Norris, 2007; see also: Arato, 2013).

In Schmitt's reasoning, the state of exception requires unrestrained sovereign authority—the *pouvoir constituant* of the people—to establish an identity-constituting foundation for the political community. In this regard, the sovereign must be above all other social groupings and not bound by their particular interests. For Schmitt, this vital architectural pillar of constitutional law is separate and detached from the specifics of the realm of “liberal” politics or the regular legal order. In the state of exception, the sovereign authority needs to be imposed in order to establish the community that makes the legal and institutional system of democracy possible in the first place (Sartori, 1989).

This is not the place to provide a fuller interpretation of Carl Schmitt's political and constitutional thinking.^3^ Rather, for the purpose of this article, I would like to draw attention to a distinct mode of thinking about the “political” that is reflected in contemporary forms of populism. The common reference point is the sovereigntist claim to defend the integrity and viability of the political community against imminent internal and external threats. Populism's political appeal critically rests on the dramatized invocation of the friend–enemy binary as a clear distinction between friendly insiders and threatening outsiders. In essence, populist politics raises the specter of a permanent state of exception in which the voice of the people is silenced and the interests or identity of the community are perpetually compromised by corrupt or unresponsive elites. The claimed existential threat fuels the need for decisive, “sovereign” action for which populists depict the rule-based legal framework of liberal democracy as being insufficiently equipped or simply deficient. I interpret this form of existential politics in the tradition of Schmitt's reasoning as the foundation of the populist notion of the political and its way of identifying the “people” via its alleged enemies.

## The Permanent “State of Exception” as Key for Populist Political Mobilization: The Campaigns of the *Alternative for Germany*

In comparative European terms, the Federal German Republic has displayed extraordinary stability in its constitutional order and government practices (Baun, 2016). A fundamental element of this stability has been the “firewall” against the far right after the horrors of the Third Reich and the Holocaust. Yet, the rise of the AfD has shattered this broad political consensus and emboldened a radical opposition against foundational rules and principles of liberal democracy. Founded in 2013, the AfD is a relatively new political force in German politics that in the 2017 general elections became the first right-wing, populist party in Federal Parliament after World War II (Art, 2018). The AfD started as a breakaway group from the conservative Christian Democrats driven by the opposition against the introduction of the Euro and the process of European integration more broadly. Over the past years, the AfD has moved decisively to the right, endorsed an aggressive anti-immigrant platform, and gradually adopted nativist ideas to its political repertoire (Decker, 2016; Lees, 2018). It is worth pointing out that the nativist ideology of the AfD is particularly rooted in Germany's Eastern regions. A factor prodding the AfD's right-wing, anti-immigrant position is related to the party joining ranks with PEGIDA, the *Patriotic Europeans Against the Islamisation of the West* movement, which is built on strong anti-Islam, anti-migrant sentiments (see: Vorländer et al., 2018). With this shift in its political identity and corresponding radicalization, the AfD now is a right-wing, populist opposition to Germany's established political parties and institutions (while at the same time being represented in national, European, and various regional Parliaments).

The AfD is such a fascinating case study for the populist vision of popular sovereignty as it seeks to reconcile two roles in Germany's democratic system. On the one hand, this party has taken up the position of the right-wing populist opposition against, what the AfD depicts as, the political establishment that betrays the true interests of the “people.” It is no coincidence that in recent campaigns against COVID-19-related public health measures, the AfD has appropriated the slogan “We are the people” originally used by the 1989 demonstrations against the Communist regime in the former German Democratic Republic. On the other hand, the AfD describes itself as operating firmly on the foundation of Germany's constitutional order and defending its key legal principles. Indeed, citing the urgent task to guard the country's sovereignty, the AfD claims to be true guardian of Germany's Constitution.^4^

The following analysis of the AfD's political campaigns is based on a discourse analysis of its public campaigns. In a qualitative research tradition, this article analyses framing practices as an interpretative lens into how an actor defines political issues and mobilizes them in political campaigns.^5^ The study assembled materials from the AfD's political campaigns analyzing speeches, parliamentary debates, party platforms, as well as electoral and social media campaigns. Altogether 429 documents (135 for 2015; 117 for 2018, and 177 for 2021) were collected and coded. First, I evaluated the documents for the presence of a dominant thematic focus and narrative structure. Second, and with a more interpretive approach, I evaluated the documents focusing on how the “will of the people” and the “enemies of the people” are framed politically. The first coding of the textual data identified on dominant themes, the second focused on the framing strategies in the political communication of the AfD.

**Figure 1.**
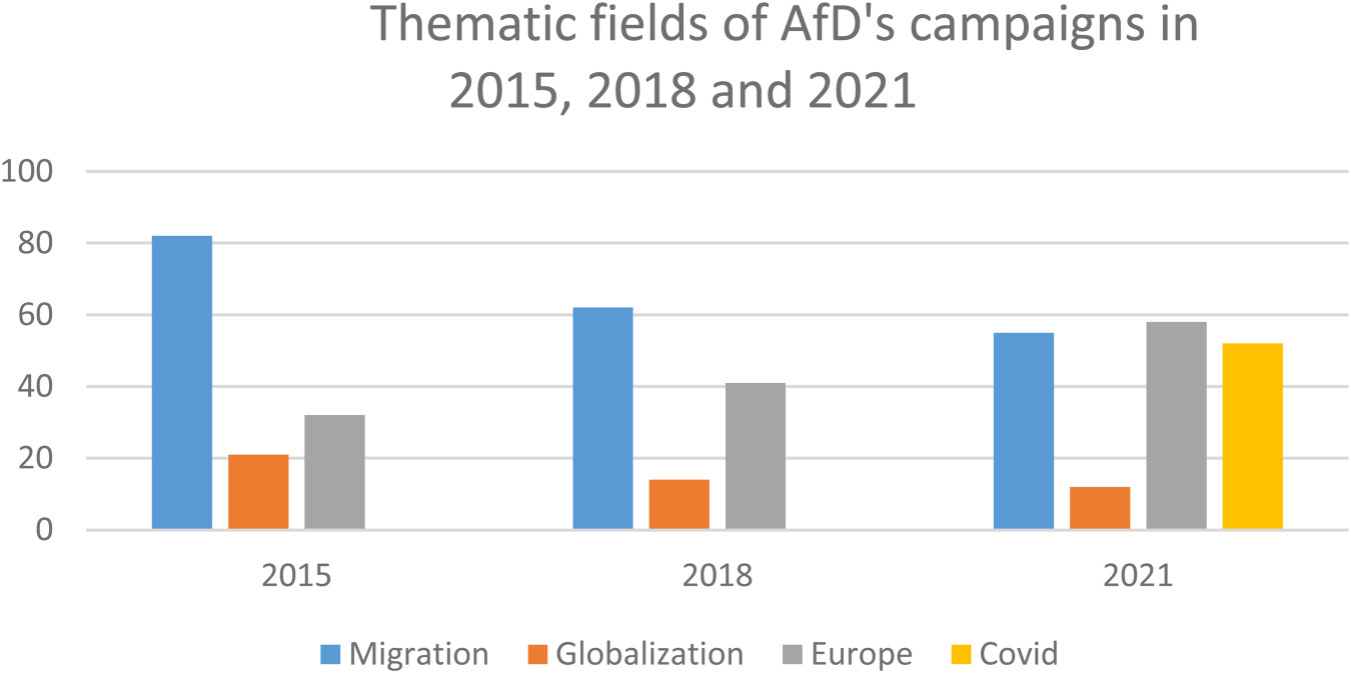
Thematic fields of *Alternative for Germany* 's campaigns in 2015, 2018, and 2021.

I categorized these iterations of the AfD's political mobilization according to key thematic fields in three distinct periods in German politics (2015, 2018, and 2021).^6^ The political issues that were most prominently addressed in the party's campaigns and that articulated the “sovereign will of the people” with the greatest degree of significance were: (a) migration and borders, (b) globalization and economic deprivation, (c) European integration and the EU, and (d) the global coronavirus pandemic. The following graph describes the relative prominence of these themes at three periods of the party's recent political engagement (Figure 1).

Considering how strongly the four themes appeared in the AfD's campaign in all three of the periods, it is striking that issues related to migration and borders have been a persistent key concern for this party. Although the issues of irregular refugees and migration no longer captures the public's political attention as it did some years ago (2015/2016 marked the height of the so-called “refugee crisis”), this issue still is in the center of the party's political mobilization. Issues related to European integration and the EU have gradually increased in relative importance and now form a central pillar of the AfD's campaigns. In contrast, issues focused on globalization and the attributed economic uncertainty are only of lesser importance for the AfD political campaigns and public discourse. Given the centrality of the COVID-19 debate, the global pandemic and the responses to related health emergencies made up an important dimension of the party's political campaigns in 2021.

In a second step, I investigated the four thematic fields with a view to how the AfD framed the respective issue as one related to which the “will of the people” needed to be restored and that is in urgent need of radical political reform. The coding of the text was guided by the following questions: How is the central political controversy framed? Who is identified as the adversary or enemy of the people? What are the ways in which the AfD suggests addressing the respective grievances? This more detailed textual analysis is meant to shed light on how the plea for “popular sovereignty” is articulated through controversial public debates on key issues where, in the interpretation of the populist party, the elite betrays the genuine interests of the people and where the *vox popoli* needs to be reinstated.

### Migration and Borders: Protecting the Us Against External Threats

Many political commentators have argued that the rise and electoral successes of the AfD are directly tied to the political fallout of the so-called European “refugee crisis” or “migration crisis” (Rensmann, 2018; Schmidtke, 2020). The AfD found its core mobilizing issue and defining mark of its political identity by opposing Chancellor Merkel's welcoming policy towards refugees in 2015/2016. At the time, her government's decision not to close the border to irregular migrants brought over one million refugees to Germany during this period alone. In policy terms, the AfD is committed to reducing the number of immigrants dramatically and abolishing Germany's constitutionally guaranteed right to asylum. While in some programmatic statements the AfD nominally endorsing a restricted version of Canada's targeted immigration policy, in its political campaigns the party has articulated a strict anti-immigration agenda. Its former parliamentary leader, Alice Weidel, stated that the AfD intends to achieve “negative immigration” and that the party's main mandate is to prevent the “Islamization” of Germany and to defend traditional Christian values. The AfD also supports strictly enforced deportation measures directed at rejected refugee applicants.

Yet, a close inspection of the political campaigns targeting immigration and border control underlines that it is not only the contested field of public policy making which is of central importance to the AfD. Immigrants or “foreigners” in general, and refugees in particular, are depicted as an existential threat to the wellbeing or even very existence of Germany. For instance, the AfD regularly links migrants to crime and social decay. When the AfD pleads for strict border controls, it mobilizes the fundamental populist rationale: The influx of migrants creates an existential crisis for the German “people”; its identity is proclaimed to be compromised and its interests betrayed. As for other right-wing populist parties, borders have become a highly productive reference point in their dramatized account of the existential risk facing their own political community. Borders provide the narrative plausibility and, in a dramatized fashion, a territorialization and visualization of what the AfD depicts as the “loss of sovereign rights” (Osuna, 2022).

The alleged lack of control over national or European borders—illustrated by large number of irregular migrants or at least the prospect of massive border crossings—has become the master frame of the populist claim that the elites have betrayed the “people.” In their narrative, national borders have the primary role as symbolic boundary markers for the “virtuous and homogeneous people.” For the AfD, the actual management of cross-border mobility as a policy challenge—the control mechanisms or resources put into place—is of lesser concern. Rather, it is the performative act of imagining a “bordered people” exposed to an existential menace from outside that takes center stage in the AfD's campaigns. Threatening images of uncontrolled masses of immigrants and the alarming presence of Muslims are dominant features in the party's campaigns. In this discursive context, the AfD presents the border as the locus at which, interchangeably, compromises the integrity of the German nation and European civilization.

Here an important difference between populist nationalist reasoning becomes apparent: Decisive for the populist campaign is the reference to the threatening other rather than the affirmative reference to what actually constitutes the people. Brubaker captures one dimension of this populist discursive strategy by his concept of “civilizationalism” (Brubaker, 2017). According to his interpretation, the populist confrontation of the threatening other is not restricted to the defense of the nation-state. It is also easily compatible with narratives of civilizational (European) superiority in particular vis-à-vis the Islamic world.

In this vein, the anti-immigrant rhetoric is both a campaign strategy to direct the frustration with a socio-economic and political reality and a way to nurture the common identity of the “people.” Migrants and foreigners are used as easy scapegoats on which the ills of society such as social decline or unemployment can be blamed. In addition, the notion of protecting their own community from “outsiders” allows for a strategically fertile projection of virtue onto their own community. Similar to other populist parties in Western democracies, the AfD is skillful in mobilizing images of a wholesome past (“*Make Germany Great Again*”) while leaving the nostalgically depicted better history vague, albeit aggressively directed against those deemed outsiders. In the party's campaigns, the immigrant is the quintessential other against whom the sovereignty of the people needs to be defended. While the AfD liberally employs the rationale of nationalist, if not nativist ideologies, it rarely engages in a substantive campaign in defining what constitutes the national community in ethnic or cultural terms.

The anti-immigrant rhetoric of the AfD provides a vivid illustration of how a nativist ideology associated with an exclusionary nationalism is productively fused with an anti-elitist political trait: The collective identity based on a clear sense of ‘Us’ (the locals, ordinary people, the Germans, etc.) and ‘Them’ (the immigrants, refugees, foreigners, etc.) is critical for the mobilizing efforts of the AfD. The strong collective identity promises itself to provide a remedy against the experience of social marginalization: pride in the national community and the promise of solidarity based on a nativist identity. Salmela and von Schewe (2017) describe how, from a social-psychological perspective, right-wing populists offer a politically effective strategy to address the fear of social decline and status inconsistency. Their underlying collective identity provides an ideational avenue to transform uncertainty and fear into resentment and hatred toward the perceived enemy of the people (Bonikowski, 2017). Blaming the ethnic or cultural “other” for social ills is as emotionally exhilarating as it is politically astute. Rensmann's diagnosis that the political radicalization of the AfD is not detrimental to its popular appeal, points to how central discourses of othering and exclusionary nationalism are to the recent electoral successes of this party (Rensmann, 2018; see also: Arzheimer and Berning, 2019; Bonikowski, 2017, Schmidtke, 2020).

### Globalization: Retreat to Classical National Sovereignty

The issue of addressing the effects of globalization and the related concerns of its supporters is more challenging for the AfD to exploit for purposes of political mobilization. In particular in its initial phase, this party had a neoliberal, strictly market-oriented platform. The force behind the drive toward a national-conservative splitting from the Christian Democrats was the dissatisfaction with the introduction of the Euro and the monetary policy by the European Union. In this respect, the AfD is distinct in the European context, as it has kept some of its market-oriented economic, social, and tax policies. In contrast, right-wing populist parties in Western democracies have regularly opted for a more expansive approach to the state's governance of the economy and to welfare state interventions.

For the AfD, the emphasis on a market rationale for defending national interests proved to be only partly compatible with its populist claim to stand up for the interests of the people deprived by powerful elites. Accompanying the reliance on neoliberal principles, the populist logic of challenging the political status quo and the orientation of its clientele^7^ has also shaped the AfD's stand on this issue. Very much in line with the binary reasoning of the populist agenda, the globalization of the economy as a fundamental threat to the wellbeing of Germany has become a mainstay in its political campaigns. The sovereigntist discourse pitching the virtuous and hard-working citizens of the national community against the irresponsible and corrupt international economic elites has gradually replaced an ideological reliance on market liberalism.

Thus, in the campaigns related to the party's stand on social and economic policies, the dominant framing is one squarely focused on national interests and the perils of globalization. According to the AfD's campaigns, Germany's only involvement in the international economy or finances should be meticulously assessed based on the expected benefits to Germany and its indigenous population (see also: Bebnowski, 2015). The widespread fear of socio-economic decline among its supporters (in particular in the former Communist – Eastern – part of Germany) is addressed with a clear-cut binary reflecting the sovereigntist ideology in a Hobbesian logic: The well-protected nation-state is the only guarantor of prosperity and security (Havercroft, 2011).

In contrast, the AfD depicts the international arena as being controlled by interests harmful to the wellbeing of the national community (sometimes with overt anti-Semitic undertones; see also: Klikauer, 2019). When it comes to governing the economy and mastering the challenges of globalization, the “enemies” of the people remain diffuse: Direct responsibility is scarcely assigned; only on rare occasions, vested economic interests and multinational companies are directly mentioned (although big banks regularly appear as agents of the globalized markets; see Schmidtke, 2021). However, the vagueness in terms of the outside threats to the community related to globalization is not necessarily detrimental to the populist political cause. The promise of security and wellbeing within national boundaries is—in its suggestive simplicity—effective when aimed at an omnipresent, albeit diffuse threat from the international world.

### Europe as the Antithesis to Popular Sovereignty

Next to the issue of migration, Germany's membership in the EU has moved into the center of the AfD's political campaigns. A group of disgruntled conservatives in the Christian Democratic political milieu started the party based on a position critical of the EU's monetary policies. In line with this type of Euro-scepticism, the AfD promotes the principle of “national self-reliance” and the primacy of national political sovereignty. In most of its party platforms and political campaigns, this party calls for scaling back the authority of the EU and diminishing its role to basic forms of economic cooperation between Europe's sovereign nation-states (a plea for a “Europe of nations”).

Yet, this criticism and a position that was highly skeptical regarding the Euro has morphed into an outright rejection of the European integration and, as happened at the April 2021 party convention, a call for Germany's exit from the EU (Schmidtke, 2021). Brexit has become an appealing blueprint for the AfD and its political constituency. The radicalization of the anti-EU stand of the AfD is couched in a distinct narrative of European integration: The EU is depicted as an alienating political force (at times accused of establishing a “dictatorship”) that undermines the fundamental rights of the “people,” their sovereignty, and freedom. In essence, the AfD describes the EU as structurally unable to sustain a democratic form of governance. As the AfD states on its current election website: “Only in national states with a democratic constitution can popular sovereignty and basic rights of the citizens be lived and preserved as the core of democracy.”^8^ At the same time, the deeply unpopular Brexit has led AfD politicians to play down the scenario of taking Germany out of the EU. The EU is a convenient “enemy” for the AfD's idea of popular sovereignty; yet, in its political discourse the AfD leadership now widely shies away from proposing an outright “Dexit.”

In its political campaigns, the AfD still works with a binary that is very much in line with its populist anti-elitist impetus. The party portrays national sovereignty as the last bastion of protecting the democratic self-determination of the “people.” Irrespective of particular policy decisions, the AfD public discourse depicts the EU being guided by an alienating bureaucracy and corrupt transnational (European) elite. The party frames the project of European integration as driven by the special interests of individual states or social groups that, in an existential fashion, undermines the viability of the national political community. More specific issues (such as wasting resources, clientelism, and corruption) appear prominently in the AfD's anti-EU narrative. The overall frame is one of the European Union destroying the foundations of the political community, its identity (also through cross-border mobility and migration), and ability to articulate its fundamental interests.

### COVID-19 as a Mode of Discrediting Scientists and Journalists as “Enemies of the State”

In 2020 and 2021, the AfD's populist agenda advocating for the “popular sovereignty of the people” found a new thematic focal point in its mobilizing efforts. First, the party struggled to find a political response to the global health pandemic that could fit its foundational anti-elitist narrative. Gradually, the AfD adjusted its political voice to denounce the public health measures and campaign for a swift end to restrictions enacted to contain the coronavirus. Essentially, the pandemic-related lockdowns, mask mandates, and immunization campaigns were opposed in the name of citizens’ freedoms and right to self-determination. Here, the inside-outside binary that informs much of the populist political narrative was projected onto the citizens’ body and physical wellbeing (based on systematic doubt spread about the efficacy and safety of the public health measures, most notably the vaccines). The AfD's public discourse on this topic fuses the anxieties of individual safety and freedom with concerns for the integrity of the body politic. In this vein, the AfD presents the opposition to the public health measures of the German government in terms of an existential crisis of the political community allegedly deprived of its sanctity and security. COVID-19 provided an important new and effective dimension of the AfD's existential politics operating with the continuous insinuation of a “state of exception.”

The controversial debate on the appropriate response to the global pandemic also reveals how COVID-19 has been addressed as a health emergency in which the “people” are betrayed—for a variety of reasons—by political, scientific, and journalistic elites. Here again, a policy debate about the effectiveness and desirability of particular measures responding to the pandemic is accompanied by a palpable discourse on how the fundamental rights of the “people” are violated. In this latter respect, the AfD's campaigns accuse those shaping these responses of deliberately misleading ordinary citizens and pursuing hidden agendas. In particular, in the social media statements of AfD politicians, the frustration over the public health responses to the coronavirus is directed against representatives of the scientific community and journalists. Both groups are accused as deliberately misleading ordinary citizens, exploiting their professional status, and betraying the “real interests of the people.” For instance, at demonstrations co-organized by the AfD, journalists are regularly insulted as “enemies of the people” or as the “lying press.”^9^ In this thematic arena, the anti-elitist impulses steer the party's political campaigns and fuel the resentment against the political establishment.

The following table summarizes how the dominant thematic issues of the AfD are framed with a view to defending the “sovereign rights of the people” against an ostensible enemy posing an existential threat to the own community. It is worth noting that the discourse in these fields is not mutually exclusive and often intimately connected. For instance, in its political mobilization the AfD regularly blames the EU for betraying the economic interests of Germany or for facilitating the influx of migrants. Nevertheless, identifying these thematic fields allows for a better understanding of what drives the AfD's populist agenda, how the people and its enemies are depicted, and what solutions are proposed for the constitutional framework of the political community (Table 1).

**Table 1.** Dominant framing in *Alternative for Germany*'s political campaigns: four thematic fields.

	Migration and borders	Globalization and the economy	European Union/Europeanization	COVID-19 pandemic
Nature of existential threat/violation of popular sovereignty	Loss of collective identity and social privileges, disruption of communal life	Growing influence of global economy; loss of control in governing economy	Supra-national government as end of national sovereignty	Health, safety, and freedom of individuals and body politics
The “people,” political community whose rights or identity are violated	National community, nation-state, ethnic group	Ordinary, hard-working citizens	Members of own national political community	Citizens restricted and violated by public health measures
Elites responsible for state of exception	National and EU elites responsible for migration policy	Global economic, financial and political elites	EU bureaucracy and institutions	National political authorities, scientific experts, journalists
Suggested remedy for addressing a state of exception and re-establishing popular sovereignty	Strict control of borders and restrictions of migration, deportation of irregular migrants	Restricting international trade and making it subject to utility for nation-state; ending multilateralism	Return power to nation-states; reinstate national sovereignty; exit from the EU	Suspending public health measures; restricting impact of “experts”

Considering these thematically distinct narratives of existential threats facing the political community, who is the sovereign in the campaigns of this populist actor? Here it is important to note the centrality of conflict and threat as a driving force behind and vehicle for the political mobilization of populist actors. Very much in line with Schmitt's theoretical categories, populists claim to defend and promote the political community in its sovereign act of self-constitution. As the analysis of the AfD's political campaigns indicates, the nature of the sovereign is not pre-determined: Rather, the sovereign decision itself constitutes the boundaries and identity of this community, primarily through the conflict between “friends and enemies.” This focus raises a subtle but important difference between traditional nationalism and the nationalism of the populists. While traditional nationalists fight for the preservation of a substantive—linguistically, ethnically, or culturally defined—homogenous community, populists’ identity of the community and the sovereign it represents is more fluid and subject to the dynamics of the political conflict itself. The nationalist agenda operates based on a relatively clearly prescribed bordered community, the *demos* defined by a territory and historically rooted identity markers. In contrast, the populist notion of the “people” and the political community whose interests, if not very existence is painted as being under existential threat, varies in accordance with the nature of the community's foes.

## Conclusions

The relative strength of populist parties across Western democracy is centrally based on the claim of empowering the “sovereign people”; Koppetsch (2019: 217) speaks in this context of the populist promise of being “collectively re-sovereignized.” The plea to represent ordinary people in their antagonistic relationship with an unresponsive elite is discursively couched in strong images of community whereby people are united by a shared collective identity. The emotionally charged sense of a community nourished and staged by nationalist populists has become one of the central political weapons to challenge what they perceive to be the technocratic *modus operandi* of liberal democracies. With an appeal to principles of popular sovereignty, populists have been able to offer a captivating and politically instrumental sense of community. In the case of right-wing populism, this invocation of a unified people in whose name their charismatic leaders claim to speak has had substantial political implications in two-fold manner: first with respect to the contempt for procedural rules in the parliamentary system, if not openly authoritarian aspirations; and second with a view to the exclusionary impetus with which the community is mobilized against alleged outsiders and “enemies of the people.”

The analysis of the AfD's political campaigns underlines this prominent return of the sovereigntist narrative under the populist banner in the first part of the 21st century. The notion of “popular sovereignty” advocates the fortification of national borders and has a close affinity to the nationalist rhetoric of protecting a political community. Yet, its primary objective is to mobilize those alienated from the established constitutional order in the name of a more direct, immediate “will of the people.” In the political arsenal of populists, the invocation of “popular sovereignty” is the emotionally charged, yet unspecific call for the empowerment of the disenfranchized.

Two foundational features in the populist political identity nurture the antagonism to the traditional constitutional order. First, populist actors depend on providing a mobilizing sense of a permanent state of exception with some narrative plausibility. The urgency of the threat and the existential crisis facing the own community of the “people” is the discursive environment in which populists flourish. Accordingly, their political mobilization is geared toward feeding the friend–enemy tension that invites the imposition of “popular sovereignty” rather than the complex decision-making process of the parliamentary system. Strikingly, the criticism that Schmitt had toward the parliamentary constitutional order with a view to its alleged weakness and lack of authority when it comes to decide as a sovereign, is—in its popularized version—omnipresent in the populist political discourse.

Second, the populist claim to represent the people operates based on the understanding of a homogenous notion of the political community. Populism's binary between the corrupt elites and the virtuous, albeit powerless people rests on the idea of a foundational collective identity. In this vision, there is no room for societal division. Internally, the notion of the “people” suggests unity and equality as a promise to its followers. Externally, it identifies the enemies against whom decisive action is warranted. This guiding idea of a unified and homogenous community is key to the plea for the “re-sovereignized” people and to the affinity right-wing populism exhibits toward nativist ideologies. However, the process by which the diversity of societal groups and voices would find their expression in a new constitutional order is not a significant part of the populist narrative. The “sovereignty” and “freedom” of the people is projected *ex negativo*, fixated on its alleged enemies.

It is this notion of a permanent crisis that populist mobilization uses as the central pretext for justifying an aggressive defense of the “sovereign people” and the political community they claim to represent. In this regard, the strengthening of the “We,” its unity and political virility, against its perceived enemies becomes the existential duty of politics. The pleas for popular sovereignty reflect the disdain for the ostensible limitation or indecisiveness of parliamentary democracy. While the AfD does not present a substantive program for constitutional reform, Schmitt's concept of the political and its underlying critique of the liberal-democratic order fundamentally shape its public engagement and political aspirations. Consequently, politics based on the division of powers and an endorsement of a pluralist society appears as weakness in the mobilizing efforts of right-wing populism. The constitutional implications of this approach to politics have come into an increasingly clear focus in those countries in which right-wing populists have taken up positions of power. The normative principle of the “will of the people” is effective in spurring political mobilization in the name of democracy. Yet, in populist politics fixated on the existential defense of this people's popular sovereignty, a system of law and government that would allow for greater democratic self-rule is an elusive idea at best. In this respect, the resurgence of right-wing populism is likely to facilitate the creeping erosion of democracy rather than offering a viable avenue for its renewal.

## Acknowledgement

I would like to acknowledge the support provided by the Social Sciences and Humanities Council of Canada (SSHRC) that has made the research for this article possible. I would also like to thank the Hamburg Institute for Advanced Study where I had the privilege of being a fellow during the work on this article.

## Notes

1. See: https://www.bbc.com/news/av/world-europe-27571813 [accessed July 3, 2021].

2. This radical-transformative quality of Schmitt's concept of the political makes it also attractive for leftist versions of populist politics (Mouffe, 2018). Populism from the left is similarly not immune to the authoritarian underpinning of Schmitt's constitutional thinking (see for instance, Booth and Baert, 2018).

3. For Schmitt's critique of liberalism and its implications for democratic processes see: Cristi (1998) and McCormick (1997).

4. See: https://www.afd.de/grundgesetz/ [accessed July 2, 2021]

5. Methodologically the article relied on discourse analysis of textual material paying particular attention to the use of language, narrative tropes, and other communicative tools (Jørgensen and Phillips, 2002; Wodak and Meyer, 2001).

6. I chose these periods (with 3-year intervals) to shed light on the changing nature of the AfD's political mobilization and identity since its launch in 2013.

7. In the 2016, the Bertelsmann Foundation found that 78% of the AfD supporters were afraid of the growing international interconnectedness of the international economy (see: https://www.sueddeutsche.de/politik/studie-der-bertelsmann-stiftung-was-die-anhaenger-rechtspopulistischer-parteien-umtreibt-1.3273071; accessed on September 18, 2021.

8. See: https://www.afd.de/europa-eu/ (accessed on July 24, 2021; own translation).

9. For some historic context it is worth pointing out that the term “lying press” (*Lügenpresse*) was prominently used by Hitler's Nationalist Socialists as a propaganda tool to discredit the independent press (see: Levi and Rothberg, 2018).
